# Dopaminergic Dysregulation in Prefrontal Cortex of Rhesus Monkeys Following Cocaine Self-Administration

**DOI:** 10.3389/fpsyt.2013.00088

**Published:** 2013-08-21

**Authors:** Scot McIntosh, Leonard Howell, Scott E. Hemby

**Affiliations:** ^1^Department of Physiology and Pharmacology, Wake Forest University School of Medicine, Winston-Salem, NC, USA; ^2^Center for Neurobiology of Addiction Treatment, Wake Forest University School of Medicine, Winston-Salem, NC, USA; ^3^Division of Neuropharmacology and Neurologic Diseases, Yerkes National Primate Research Center, Atlanta, GA, USA; ^4^Department of Psychiatry and Behavioral Sciences, Emory University School of Medicine, Atlanta, GA, USA; ^5^Department of Psychiatry and Behavioral Medicine, Wake Forest University School of Medicine, Winston-Salem, NC, USA

**Keywords:** cocaine, dopamine, orbitofrontal cortex, anterior cingulate cortex, dorsolateral prefrontal cortex, rhesus monkey

## Abstract

Chronic cocaine administration regulates the expression of several proteins related to dopaminergic signaling and synaptic function in the mesocorticolimbic pathway, including the prefrontal cortex. Functional abnormalities in the prefrontal cortex are hypothesized to be due in part to the expression of proteins involved in dopamine signaling and plasticity. Adult male rhesus monkeys self-administered cocaine (i.v.) under limited (*n* = 4) and extended access conditions (*n* = 6). The abundance of surrogate markers of dopamine signaling and plasticity in the dorsolateral prefrontal cortex (DLPFC), orbitofrontal cortex (OFC), and anterior cingulate cortex (ACC) were examined: glycosylated and non-glycosylated forms of the dopamine transporter (efficiency of dopamine transport), tyrosine hydroxylase (TH; marker of dopamine synthesis) and phosphorylated TH at Serine 30 and 40 (markers of enzyme activity), extracellular signal-regulated kinase 1 and 2 (ERK1 and ERK 2), and phosphorylated ERK1 and ERK2 (phosphorylates TH Serine 31; markers of synaptic plasticity), and markers of synaptic integrity, spinophilin and post-synaptic density protein 95 (roles in dopamine signaling and response to cocaine). Extended cocaine access increased non-glycosylated and glycosylated DAT in DLPFC and OFC. While no differences in TH expression were observed between groups for any of the regions, extended access induced significant elevations in pTH^Ser31^ in all regions. In addition, a slight but significant reduction in phosphorylated pTH^Ser40^ was found in the DLPFC. Phosphorylated ERK2 was increased in all regions; however, pERK1 was decreased in ACC and OFC but increased in DLPFC. PSD-95 was increased in the OFC but not in DLPFC or ACC. Furthermore, extended cocaine self-administration elicited significant increases in spinophilin protein expression in all regions. Results from the study provide insight into the biochemical alterations occurring in primate prefrontal cortex.

## Introduction

Cocaine abuse is characterized in part by short-term and long-term neuroadaptations in brain regions that affect subsequent intake, withdrawal, relapse, and cognition. The majority of non-human research conducted in this area has focused on alterations in the mesolimbic dopamine system that mediates motivational processes (1). More recently, the involvement of prefrontal cortical regions in cocaine addiction has received increasing attention. The prefrontal cortex is generally regarded as a principle neural substrate of executive cognition (2–8). The dorsolateral prefrontal cortex (DLPFC) is generally associated with planning, organizing, and modifying motor output, sensory integration, and working memory, the orbitofrontal cortex (OFC) with sensory integration, reward salience and adaptive learning, and the anterior cingulate cortex (ACC) with error detection, attention, and regulation of emotional responses.

Several studies clearly demonstrate prefrontal cortical alterations following chronic cocaine use that manifest as changes in various cognitive deficits. Cocaine addicts exhibit deficits in behavioral inhibition and attentional shifting that are qualitatively similar to individuals who have suffered damage to prefrontal regions. Abstinent cocaine users exhibit several impairments of cognition that are associated with functional alterations in the prefrontal cortex including (1) impairment of decision making and executive control associated with increased activation of the OFC (9, 10), (2) impairment of visuo-spatial working memory and sustained attention associated with decreased activation of the anterior cingulate and DLPFC (11, 12), (3) increased delayed discounting associated with decreased activation of the prefrontal and anterior cingulate cortices (13), (4) increased reactivity to audio-visual cues associated with activation of the anterior cingulate and dorsolateral prefrontal cortices (14–17), and (5) lower sensitivity to rewards associated with decreased activation of the orbitofrontal and prefrontal cortex (18). The neurobiological alterations in conjunction with the associated cognitive deficits likely undermine the ability of the cocaine user to abstain from subsequent drug intake and may serve as biological impediment to treatment. Thus, understanding the long-term effects of cocaine use is critical to developing medicinal and psychotherapeutic interventions that will attenuate or possibly reverse the neuropathological effects of cocaine.

Current understanding of stimulant-induced neurobiological alterations (including expression and functional genomics/proteomics, epigenetic modifications, etc.) is based predominantly on rodent models of human drug intake. The direct determination of protein expression status and factors regulating protein expression in *primate* brain have been lacking, yet are essential for understanding the neuropathology of cocaine use in humans. The use of a non-human primate model of cocaine abuse enables a clear and clinically relevant characterization of behavioral and biochemical changes associated with chronic cocaine use in a well-controlled laboratory setting. Moreover, non-human primate cortex exhibits significant anatomical and biochemical similarities with human cortex that facilitate the translation of findings to humans (19–22).

Dopaminergic signaling within the mesocorticolimbic pathway serves as a principal substrate for the reinforcing effects (REFS) of abused drugs and is critically involved in cognition and information processing (23–28). Dopamine afferents are found in high densities in the OFC, DLPFC, and ACC) (29, 30) arising from the dorsal aspects of the three mesencephalic dopamine cell groups (31). The origins of the projections and their topography are essentially the same in macaque monkey and human (32).

The present study characterized fundamental biochemical adaptations in prefrontal cortical brain regions of rhesus monkeys following limited access and extended access conditions of cocaine self-administration by assessing the expression of specific markers of dopamine signaling and dopamine mediated plasticity. The dopamine transporter is the principal mechanism for removing dopamine from the synaptic cleft and a primary target for cocaine’s REFS. DAT activity is dependent upon the protein’s glycosylation status, with glycosylated DAT more abundantly expressed at the cell surface and more efficient in transporting dopamine (33, 34). Previous studies have demonstrated elevated DAT protein levels in the prefrontal cortex following chronic cocaine self-administration in rats (35); however, no studies to date have assessed glycosylation states of DAT following cocaine self-administration. We hypothesize glycosylated DAT levels will be increased following cocaine self-administration. Native and phosphorylation states of tyrosine hydroxylase (TH) will also be assessed. TH is the rate-limiting enzyme in dopamine synthesis and therefore the principal regulator of cytosolic dopamine levels. Cocaine-induced regulation of dopamine is well documented in the ventral tegmental area and the nucleus accumbens in rodent models with increases, decreases or no change reported depending on the dosing and treatment regimen (36–41). In the rodent PFC, TH levels are increased following experimenter administered cocaine (36), while levels are not changed following cocaine self-administration (35). Phosphorylation of TH serine residues 31 (pTH^Ser31^) and 40 (pTH^Ser40^) increase the enzyme’s activity. Cocaine self-administration has been shown to increase pTH^Ser31^ (42) and decrease pTH^Ser40^ levels in rodent striatum (43). We hypothesize no change in native TH levels, increased pTH^Ser31^ and decreased pTH^Ser40^ levels following cocaine self-administration. Extracellular signal-regulated kinases 1 and 2 (ERK1/2) are involved in cocaine-induced synaptic plasticity (44) and mediate the phosphorylation of TH at Serine 31 (45). We hypothesize that increases in pTHSer31 will be accompanied by concomitant increases in the phosphorylation of ERK1 and ERK2. In addition, we hypothesize increased levels of spinophilin, a synaptic scaffolding protein in dendritic spines, and decreased levels of post-synaptic density protein 95 (PSD-95), a scaffolding protein located in the post-synaptic density, as the name indicates. Both proteins play distinct and important roles in dopamine signaling and responses to psychomotor stimulant administration.

## Materials and Methods

### Surgery and self-administration procedures

Eighteen male adult rhesus monkeys (*Macaca mulatta*) were singly housed with standard enrichment, including social enrichment, human interaction, variety in diet, and age appropriate objects as dictated by the Animal Welfare Act and the Emory University Policy for Environmental Enhancement. Food and water were available *ad libitum*. Animal care procedures strictly followed the National institutes of Health Guide for the Care and Use of Laboratory Animals and were approved by the Institutional Animal Care and Use Committee of Emory University School of Medicine.

Ten subjects were surgically prepared with chronically indwelling venous catheters using procedures described previously (46). Under appropriate anesthesia, either isoflurane alone or ketamine in combination with diazepam, and under aseptic conditions, one end of a silicone catheter was passed by way of a jugular or femoral vein to the level of the right atrium and vena cava, respectively. The distal end of the catheter was passed under the skin and attached to a vascular access port (Access Technologies, Skokie, IL, USA) that remained subcutaneous in the center of the back for easy access. The 0.25 ml unit was accessed during testing with special right-angle Huber needles (Access Technologies, Skokie, IL, USA) that minimize damage to the port membrane and allow for repeated punctures over a year or more. Catheters were flushed periodically with heparinized saline (0.9%). Daily experimental sessions were conducted within a ventilated, sound-attenuating chamber with each monkey seated in a standard primate chair of the type commercially available (Primate Products, Redwood City, CA, USA). A panel equipped with a response lever and stimulus lights was mounted on the front of the chair. The vascular access port was connected via polyvinyl-chloride tubing to a motor-drive syringe located outside the test chamber to yield a precise injection volume of 2.0 ml during drug self-administration experiments with dose determined by the concentration of drug solution in the syringe. Experimental procedures were controlled on-line by a microprocessor and electromechanical programing systems, and data were monitored and recorded during daily sessions.

One group of four rhesus monkeys comprised the limited access condition and was trained to self-administer cocaine by pressing a response key while seated in a primate chair. Responding was initiated using a 1-response fixed-ratio schedule (FR 1) so that each response in the presence of a red light produced an intravenous drug injection and the brief illumination of a white light followed by a timeout. The ratio value was increased gradually as responding increased. When the schedule value reached FR 20, drug injection no longer followed completion of each FR and, instead, was arranged to follow an increasing number of FR components. Ultimately, the schedule was a second-order schedule of FR 20 components with drug injection following the first component completed after 10 min had elapsed [FI 600 s (FR 20:S)]. A 2-s white light was presented upon completion of each FR 20 component. Drug administration was accompanied by a change in the stimulus light from red to white for 15 s, followed by a 1-min timeout. Daily sessions consisted of five consecutive 10 min intervals. The unit dose of cocaine remained constant at 0.1 mg/kg/injection (46, 47). Use of this second-order procedure and limiting the daily session to approximately 1 h enabled the standardization of total drug intake to 0.5 mg/kg/session. Previously, we have reported significant alterations in ionotropic glutamate receptor subunits in the NAc from the limited access subjects used in the present study (20).

A separate group of six monkeys was trained as described above and composed the extended access group. The initial limited access condition as described above was then followed by 3 months of extended access conditions during which monkeys had an opportunity 3 days per week to increase the amount of cocaine self-administered. On extended access days, sessions began with the second-order schedule, followed by two additional hours on FR 20 schedule for total session duration of 3 h. During the additional 2 h of extended access, a green stimulus light illuminated the chamber and each FR 20 completed resulted in the presentation of the 15-s white light and a 0.1 mg/kg infusion of cocaine. Each infusion received was followed by a 60-s timeout. Monkeys had the opportunity to take an additional 30 infusions (3.0 mg/kg total) during the extended access condition in addition to the 0.5 mg/kg available during the 1 h second-order schedule. Behavioral data for the monkeys in the extended access group have been reported previously (48).

### Tissue dissection

Following completion of the drug self-administration studies, monkeys were restrained with Telazol, given intravenous heparin and then an overdose of intravenous sodium pentobarbital. Four control monkeys were euthanized as described above. After the confirmed absence of brain stem reflexes was established, the monkeys were transcardially perfuse with ice cold phosphate buffered saline (PBS; pH 7.2–7.4), the brain was removed and placed in 4°C PBS for 5 min.

Following euthanasia and removal of the brain, the prefrontal cortex was dissected from the rest of the brain and the OFC (Area 13), ACC (Area 24), and DLPFC (Area 46) were dissected as described previously (49). The OFC dissection included the medial and lateral aspects of the medial orbital sulcus located rostral to the rostral end of the corpus callosum, and therefore included Areas13a and 13m (50). The ACC was dissected from the rostral pole of the corpus callosum and included tissue from the cingulate gyrus up to but not including the lower bank of the cingulate sulcus, containing fields 24a and 24b. The DLPFC (Area 46) was dissected from the banks of the principle sulcus midway along its length.

### Protein isolation and fractionation

A steel mortar and pestle chilled in dry ice were used to pulverize the frozen brain tissue into a dry homogenate in the presence of liquid nitrogen. Tissue proteins were fractionated into membrane, nuclear, and cytosolic fractions as described previously (20, 22, 51, 52). Protein was quantified using the bicinchoninic acid protein assay kit (Pierce Biotechnology) and equivalent final protein concentrations of each sample were diluted in Laemmli sample buffer, boiled at 95°C for 5 min, and subjected to electrophoresis. Samples were separated on Tris-HCl SDS-PAGE gels and transferred to nitrocellulouse membranes. Membranes were blocked with Odyssey blocking buffer (Licor, Lincoln, Nebraska) and incubated with primary antibody overnight at 4°C. Primary antibodies included anti-dopamine transporter (Millipore, #AB2231, 1:1000), anti-TH (Sigma, #T1299, 1:5000), anti-phospho-TH (pSer^31^) (Sigma, #SAB4300674, 1:1000), anti-phospho-TH (pSer^40^) (Sigma, #T9573, 1:1000), anti-ERK1/2 (Cell Signaling Technology, #4695, 1:1000), anti-phospho- ERK1/2 at Thr202/204 (Cell Signaling Technology, #4370, 1:1000), anti-spinophilin (Millipore, #06-852, 1:1000), and anti-PSD-95 (Millipore, #MAB-1596, 1:1000). Antibodies were visualized with fluorophore conjugated secondary antibodies. Blots were scanned with the Licor Odyssey infrared scanner and signals were quantified with Odyssey software. Equal protein loading and efficiency of transfer were confirmed by probing blots with a GAPDH antibody (Millipore, #MAB-374, 1:500). Signal intensities for proteins of interest were reported as percent control relative to GAPDH.

### Data analysis

Background-subtracted intensity values for each sample were calculated using Odyssey version 1.2 software. Data from each region were analyzed using one way analysis of variance with treatment as the factor and hybridization intensity relative to GAPDH as the dependent variable. *Post hoc* analysis was performed using Tukey’s test and the null hypothesis was rejected when *P* < 0.05.

## Results

### Behavioral data

Once stable self-administration behavior was established under the second-order schedule, subjects reliably obtained all scheduled injections during daily sessions. Cocaine self-administration for subjects in the limited access and extended access groups have been reported previously (20, 48). The average total intake was 37.9 ± 4.6 mg/kg for the limited access group and 151.8 ± 24.3 mg/kg for the extended access group. For the limited access group, the average session intake was 0.46 ± 0.1 mg/kg for the final three months of stable self-administration. For the extended access group, the average session intake was 1.81 ± 0.5 mg/kg during the three months of extended access.

### Western blot

Western blot analysis was performed on membrane (DAT) or cytosolic protein fractions from individual monkeys in the Limited Access, Extended Access, and control groups to assess levels of cocaine-regulated proteins.

#### Dopamine transporter

**Figure 1:**
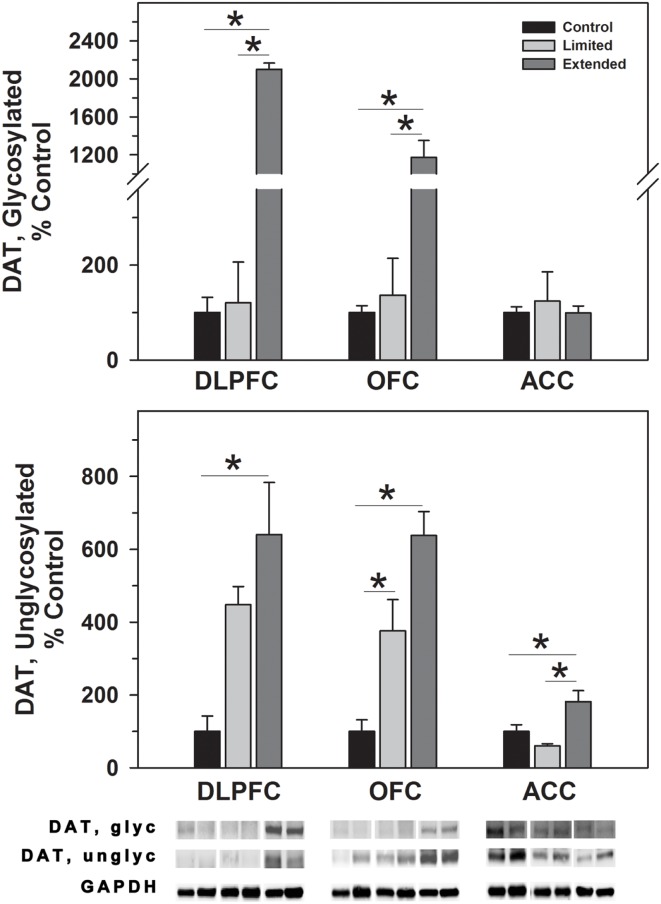
**Western blot analysis of glycosylated and non-glycosylated dopamine transporter protein levels in prefrontal regions following limited and extended access to cocaine self-administration**. Membrane fractions were isolated as described in the Section “Materials and Methods” and 15 μg were separated on 10% SDS-PAGE. Data are expressed as mean (±SEM) of the percent of control values per amount of protein loaded. Asterisks indicate a significant difference (*P* < 0.05). Bottom Panel: Representative bands from two subjects per group per region.

In the DLPFC, glycosylated dopamine transporter levels were significantly increased in [*F*(2,17) = 24.92, *P* < 0.001] in the Extended Access Group compared to Limited Access and Controls. Similarly, non-glycosylated DAT levels were increased in the extended access group compared to controls, but not to Limited Access [*F*(2,17) = 5.334, *P* = 0.018]. Similar to the DLPFC, glycosylated DAT levels in the OFC were significantly increased in the Extended Access Group compared to Limited Access and Controls in the OFC [*F*(2,17) = 44.44, *P* < 0.001]. Un-glycosylated DAT levels in the OFC were increased in the extended access group compared to Limited Access, and between Limited Access and controls [*F*(2,17) = 25.072, *P* < 0.001]. No changes were observed in levels of glycosylated DAT in the ACC; however, non-glycosylated DATA levels were significantly greater in the Extended Access group compared to Limited Access and controls [*F*(2,16) = 7.55, *P* = 0.006] (Figure 1).

#### Tyrosine hydroxylase

**Figure 2:**
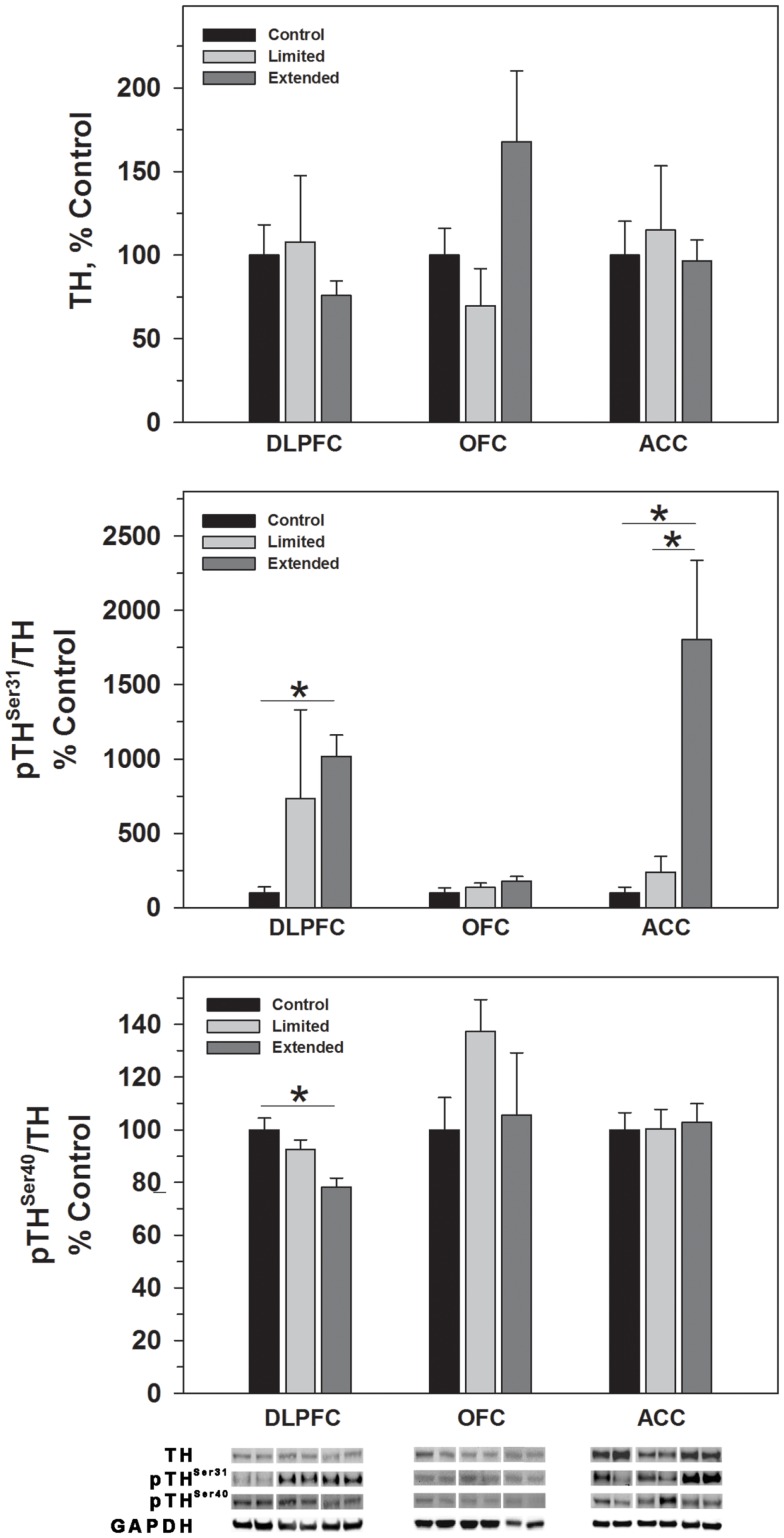
**Protein levels of TH, pTH^Ser31^, and pTH^Ser40^ in prefrontal cortical regions as a function of cocaine self-administration exposure determined by Western blot analysis**. Cytosolic fractions were isolated as described in the Section “Materials and Methods” and 10 μg were separated on 10% SDS-PAGE. Data are expressed as mean (±SEM) of the percent of control values per amount of protein loaded. Asterisks indicate a significant difference (*P* < 0.05). Bottom Panel: Representative bands from two subjects per group per region.

**Figure 3:**
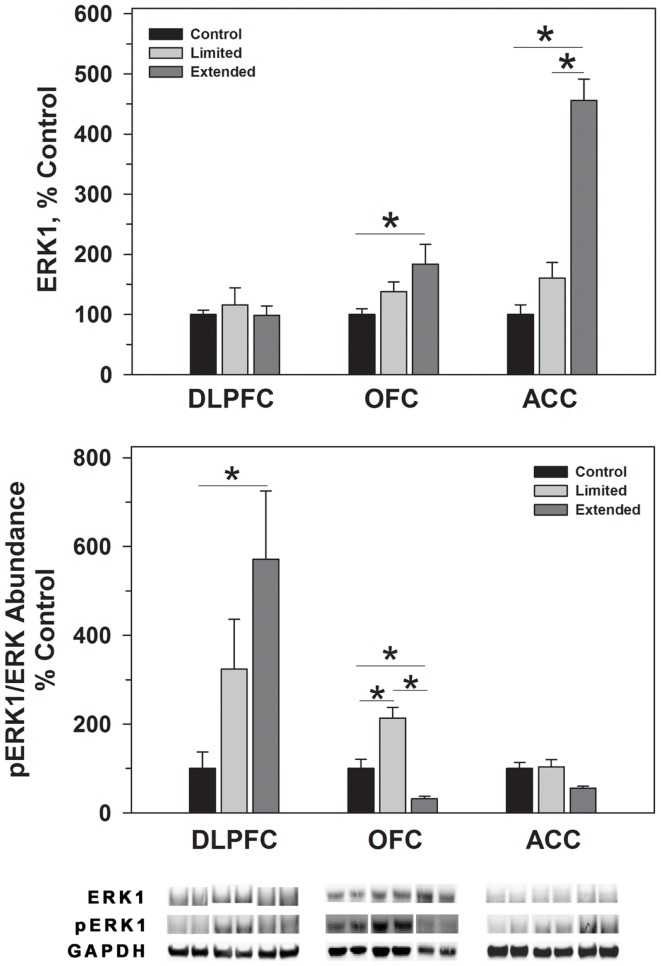
**Protein levels of ERK1 and pERK1 in prefrontal regions following limited and extended access to cocaine self-administration**. Cytosolic fractions were isolated as described in the Section “Materials and Methods” and 10 μg were separated on 10% SDS-PAGE. Data are expressed as mean (±SEM) of the percent of control values per amount of protein loaded. Asterisks indicate a significant difference (*P* < 0.05). Bottom Panel: Representative bands from two subjects per group per region.

While no significant difference was observed in TH levels, TH phosphorylated at Serine 31 (pTH^Ser31^) was elevated in the Extended Access group compared to Controls [*F*(2,17) = 4.754, *P* = 0.025] (Figure 2). Conversely, phosphorylation of serine 40 on TH (pTH^Ser40^) was significantly decreased in the Extended Access group compared to Controls [*F*(2,17) = 8.86, *P* = 0.003] (Figure 2). In the OFC, no significant differences were observed in TH, pTH^Ser31^, or pTH^Ser40^ between the groups (Figures 2 and 3). Similarly, no significant differences were observed for TH or pTH^Ser40^ between the groups in the ACC. Levels of pTH^Ser31^ were significantly increased in the Extended Access group compared to Limited Access and controls [*F*(2,17) = 11.30, *P* < 0.001] (Figure 2).

#### Extracellular signal-regulated kinase

**Figure 4:**
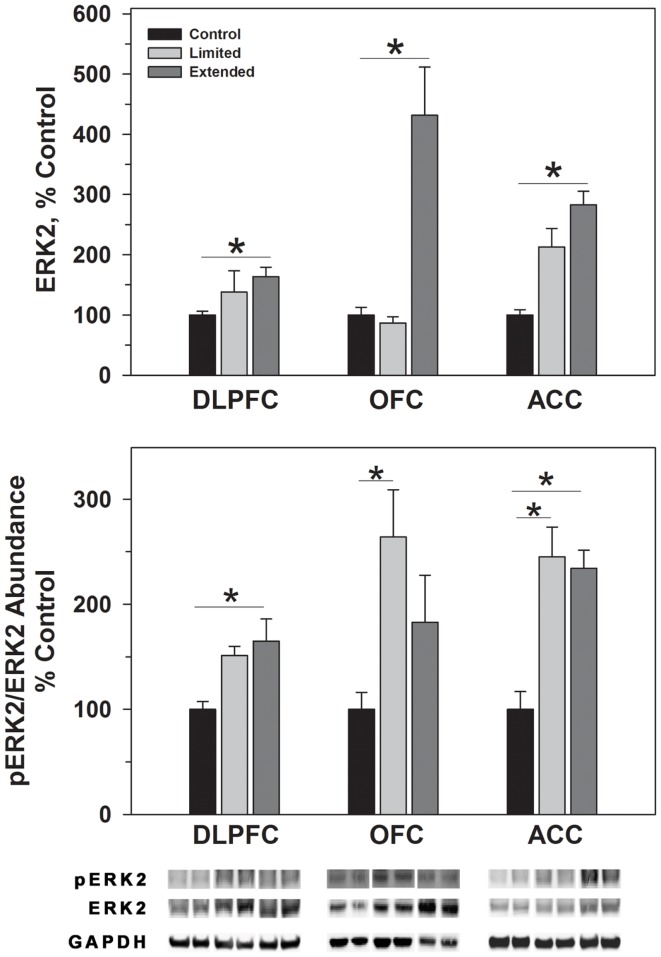
**Protein levels of ERK2 and pERK2 in prefrontal regions following limited and extended access to cocaine self-administration**. Cytosolic fractions were isolated as described in the Section “Materials and Methods” and 10 μg were separated on 10% SDS-PAGE. Data are expressed as mean (±SEM) of the percent of control values per amount of protein loaded. Asterisks indicate a significant difference (*P* < 0.05). Bottom Panel: Representative bands from two subjects per group per region.

ERK1 and ERK2 phosphorylate TH^Ser31^ among other proteins. Whereas no changes were observed for ERK1 in the DLPFC region, phosphorylated ERK1 levels were significantly greater in the Extended Access group compared to controls [*F*(2,17) = 6.307, *P* = 0.01]. Extended Access to cocaine-induced a significant increase in ERK2 [*F*(2,17) = 5.623, *P* = 0.015] as well as phosphorylated ERK2 levels [*F*(2,17) = 6.40, *P* = 0.007] over control values (Figure 4).

In the OFC, Extended Access to cocaine-induced a significant increase in ERK1 [*F*(2,17) = 5.623, *P* = 0.015] compared to control values. However, pERK1 levels were increased following Limited Access compared to control levels, and significantly less following Extended Access compared to Limited Access [*F*(2,17) = 18.605, *P* < 0.001]. ERK2 levels were greater in the Extended Access group compared to controls [*F*(2,17) = 20.075, *P* < 0.001], while pERK2 levels were increased in the Limited Access group compared to controls [*F*(2,17) = 5.185, *P* = 0.021] (Figure 4).

In the ACC, Extended Access to cocaine-induced a significant increase in ERK1 [*F*(2,17) = 39.53, *P* < 0.001] compared to Limited Access and control values. pERK1 levels were significantly different between the groups [*F*(2,17) = 4.26, *P* = 0.034], however, *post hoc* analysis did not reveal the nature of the differences. ERK2 levels were greater in the Extended Access group compared to controls [*F*(2,17) = 6.19, *P* = 0.011], while pERK2 levels were increased in the Limited Access and Extended Access groups compared to controls [*F*(2,17) = 18.576, *P* < 0.001] (Figure 4).

#### Spinophilin and PSD-95

**Figure 5:**
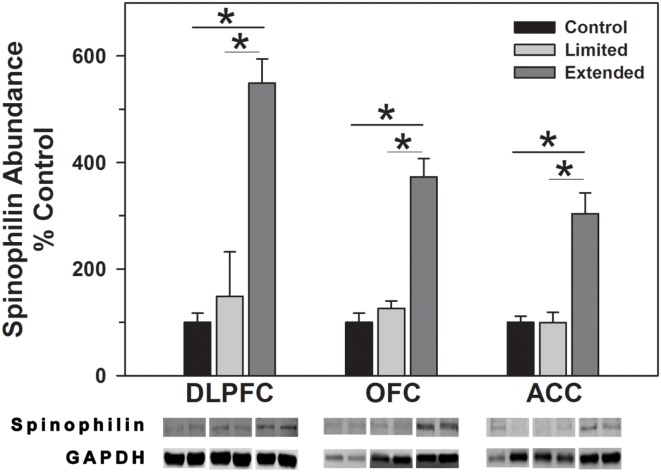
**Western blot analysis of spinophilin protein levels in prefrontal regions following limited and extended access to cocaine self-administration**. Cytosolic fractions were isolated as described in the Section “Materials and Methods” and 10 μg were separated on 10% SDS-PAGE. Data are expressed as mean (±SEM) of the percent of control values per amount of protein loaded. Asterisks indicate a significant difference (*P* < 0.05). Bottom Panel: Representative bands from two subjects per group per region.

Levels of spinophilin and PSD-95 were assessed to determine changes in dendritic spines and post-synaptic densities following chronic cocaine self-administration. In the DLPFC, levels of spinophilin were significantly increased in the Extended Access compared to Limited Access and Controls [*F*(2,17) = 44.09, *P* = 0.001]. In the OFC, spinophilin levels were significantly increased in the Extended Access compared to Limited Access and Controls [*F*(2,17) = 38.12, *P* < 0.001]. Levels of spinophilin in the ACC were significantly increased in the Extended Access compared to Limited Access and Controls [*F*(2,17) = 21.58, *P* < 0.001] (Figure 5).

**Figure 6:**
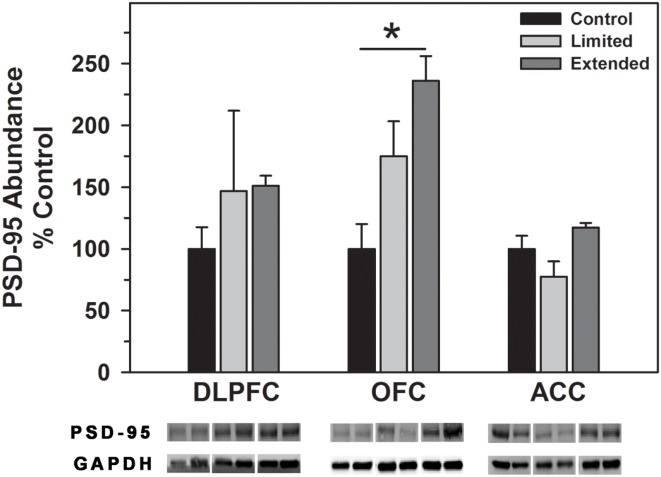
**Western blot analysis of PSD-95 protein levels in prefrontal regions following limited and extended access to cocaine self-administration**. Cytosolic fractions were isolated as described in the Section “Materials and Methods” and 10 μg were separated on 10% SDS-PAGE. Data are expressed as mean (±SEM) of the percent of control values per amount of protein loaded. Asterisks indicate a significant difference (*P* < 0.05). Bottom Panel: Representative bands from two subjects per group per region.

No significant difference in the level of PSD-95 in the DLPFC was observed between the groups. In contrast, Extended Access to cocaine resulted in increased levels of PSD-95 compared to controls in the OFC [*F*(2,16) = 11.20, *P* < 0.001], whereas no significant difference in the level of PSD-95 was observed between the groups in the ACC (Figure 6).

## Discussion

The current results indicated that cocaine self-administration up-regulated various aspects of dopamine transmission in the prefrontal cortex in a region specific manner. Significant increases in non-glycosylated and glycosylated DAT were found in the DLPFC and OFC following extended cocaine access. While no differences in TH expression were observed between groups for any of the regions, extended access induced significant elevations in pTH^Ser31^ in all regions. In addition, a slight but significant reduction in phosphorylated pTH^Ser40^ was found in the DLPFC. pTH^Ser31^ is phosphorylated by ERK1 and ERK2. Phosphorylated ERK2 was increased in all three regions; however, pERK1 levels were decreased in the ACC and OFC but increased in the DLPFC. PSD-95, which interacts with the D1 receptor and DAT, was increased in the OFC but not in the DLPFC or ACC. Furthermore, extended cocaine self-administration elicited significant increases in spinophilin protein expression in all regions. These data support and expand the results of studies in humans implicating dopaminergic dysregulation in cocaine abuse and provide molecular markers of prefrontal dysregulation in cocaine addiction.

Cocaine abuse is characterized by uncontrollable drug-seeking and drug-taking behaviors, increased use in the presence of negative consequences and inflexibility to make necessary adjustments to decrease or cease drug use (53). In general, the actions of the DLPFC, OFC, and parts of the ACC mediate behavioral control and flexibility in decision-making processes, the same prefrontal cortical areas dysregulated in cocaine addicts. Numerous studies have shown that chronic cocaine abuse results in “hypofrontality” as evidenced by decreased rates of glucose usage (4, 54, 55) and disrupted cerebral blood flow (56–58).

Deficits in specific aspects of executive function that are accompanied by changes in prefrontal function (59). For example, decreased response inhibition is accompanied by decreased activation of the ACC and DLPFC during GO-NOGO task performance in cocaine abusers compared to controls (60), while cocaine abusers exhibited poor performance on the Stroop task that was accompanied by decreased glucose utilization in the OFC (8). During tasks to assess updating activity and capacity, cocaine abusers exhibited working memory hyper-activation of the DLPFC whereas working memory load activation (capacity) of the DLPFC was significantly lower in the cocaine group compared to controls (61). When assessing the effect of memory loading on response inhibition, Hester and Garavan found that as memory load increased, response inhibition decreased and was accompanied by decreased ACC activity in cocaine abusers (62). With regard to cognitive flexibility, poor performance on attention switching tasks is associated with decreased OFC and ACC activity in cocaine abusers (9, 11). In toto, these studies suggest a neuroanatomical basis for cognitive dysfunction in addiction, supporting a prominent role that such dysfunction may play in prolonging abuse or predisposing users toward relapse.

While numerous studies have identified prefrontal cortical regions associated with cocaine-induced cognitive dysfunction, characterizing the biochemical bases of prefrontal dysregulation associated with cocaine abuse has proven to be less straightforward. In a previous study in rhesus monkeys, a challenge infusion of cocaine following limited access self-administration increased metabolic activity throughout the prefrontal cortex including the DLPFC (Area 46), rostral superior frontal gyrus (Area 11), ventral ACC (Area 24) with the most robust activation in the ventral OFC (Area 13 and 47). Extended access was shown to induce broader metabolic activity in the prefrontal cortex to include rostral prefrontal cortex (Area 10), in addition to those areas activated during limited access, with the most prominent activation in the DLPFC (63). The pattern of expanded involvement of prefrontal cortical domains as a function of cocaine exposure has also been demonstrated in macaques using the 2-[^14^C]deoxyglucose method (64–66). While metabolic activation studies have clearly shown involvement of prefrontal cortical regions in cocaine abuse, the biochemical basis for these changes remains uncertain. Given the dense dopaminergic innervation, the laminar distribution and densities of dopamine receptors in the DLPFC, OFC, and ACC and the essential modulatory role of dopamine on circuits involved in cognitive functions including working memory, response inhibition, set-shifting, and updating (23, 67, 68), it is reasonable to propose that cocaine-induced alterations in dopamine transmission and signaling may have a significant impact on prefrontal function and correspondingly on cognitive function.

The goal of the current study was to assess biochemical markers of dopaminergic transmission/signaling and synaptic plasticity in prefrontal regions following limited and extended access to cocaine self-administration in rhesus monkeys. The dopamine transporter serves as the principal mechanism for removal of the neurotransmitter from the synapse. Cocaine inhibition of dopamine uptake leads to significant elevations of dopamine in the synaptic cleft an effect to which the reinforcing and behavioral effects of cocaine are attributed (69–71). DAT function is dependent upon the glycosylation status such that increased glycosylation is related to increased membrane expression as well as increased DA uptake. Non-glycosylated DAT is present at the membrane surface and appears to be functional to some extent (33, 34). In the present study, extended cocaine self-administration access resulted in increased levels of glycosylated DAT in the DLPFC and OFC and increased levels of non-glycosylated DAT in all three regions. Comparably, DAT binding is increased in the striatum, following chronic cocaine self-administration (3 months) (72) as well as in the striata of human cocaine overdose victims (73–76). The present results demonstrate that DAT abundance in primate prefrontal cortex is sensitive to chronic blockade by cocaine and suggest DAT glycosylation is another adaptive mechanism induced by cocaine.

The enzymatic activity of TH, the rate-limiting enzyme of catecholamine biosynthesis, is regulated by phosphorylation of serine residues 31 and 40 in the N terminal regulatory domain that are phosphorylated by a variety of kinases. TH^Ser40^ is phosphorylated by CAMP dependent protein kinase and to a lesser extent by calcium-calmodulin kinase and is the primary generator of TH activity. In addition, TH^Ser40^ phosphorylation relieves feedback inhibition, in this case, from dopamine. TH^Ser31^ is phosphorylated by ERK1 and 2 and also increases TH activity but to a lesser extent than TH^Ser40^ phosphorylation (45). Also, TH activity can be regulated long term by an increase in protein synthesis, however, we did not observe a change in TH levels in any of the prefrontal regions assessed following limited or extended access cocaine self-administration, similar to findings in the PFC of rats following 10 days of self-administration (35). Instead, we observed region specific changes in the phosphorylation of TH^Ser31^ and TH^Ser40^. In the DLPFC and ACC, levels of phosphorylated TH^Ser31^ were elevated while in the DLPFC pTH^Ser40^ was decreased following extended access cocaine self-administration. Elevated pTH^Ser31^ may reflect an increase in TH activity and dopamine synthesis, albeit at a lower level of activity than pTH^Ser40^ while decreases in pTH^Ser40^ likely reflect feedback inhibition of dopamine synthesis as shown preciously in the NAc following chronic cocaine self-administration in rats (43).

The MAPK/ERK pathway is a signaling cascade that is activated by neurotrophic factors and regulated by the Ras family of GTPases. Activation of the pathway regulates the phosphorylation of various proteins, including TH^Ser31^ (45), as well as transcription and translation of various molecules involved in cognitive processes (77) and long-term synaptic changes (78–81). In the present study, phosphorylation of TH^Ser31^ was increased following extended cocaine access that was accompanied by concomitant changes in pERK1/ERK1 and pERK2/ERK2 levels in the DLPFC. While similar increases were observed in pTH^Ser31^ in the ACC, there was a trend toward decreased pERK1/ERK levels and a significant increase in pERK2/ERK2 levels following extended access. Previous studies have indicated that ERK1 is poorly phosphorylated in the brain and therefore possesses little direct signaling capacity *in vivo* (80). Furthermore, ablation of ERK1, as in ERK1 knockout mouse, resulted in a stimulus dependent increase in ERK2 signaling due to an enhanced interaction with MEK (79). Reduction of pERK1 and elevation of pERK2 suggests that TH^Ser31^ phosphorylation is being driven primarily through ERK2 activity in the ACC. Interestingly, increased ratios of pERK1/ERK1 and pERK2/ERK2 in the limited access group did not correlate with increased TH^Ser31^ levels in the OFC. The differences in ERK-dependent signaling responses amongst the DLPFC, ACC, and OFC are interesting but not unexpected as a previous study demonstrated regions specific changes in ERK-dependent signaling in the accumbens, hippocampus, and amygdala (79).

Synaptic plasticity is regulated by the expression and modifications of a variety of post-synaptic proteins, such as spinophilin and PSD-95. Spinophilin is highly enriched in the majority of dendritic spines and regulates the morphology and function of dendritic spines (82). PSD-95 is a post-synaptic scaffolding protein that is involved in the maturation and stability of excitatory synapses (83). As such, spinophilin and PSD-95 serve as proxies for changes in the abundance, size, and shape of dendritic spines and excitatory synapses in the brain, respectively. Alterations in the abundance of these proteins has been associated with changes in cognition (84, 85) synaptic plasticity and development (86–89) as well as cocaine exposure (90). In the present study, extend access cocaine self-administration resulted in elevated levels of spinophilin in all three cortical regions, while PSD-95 was only increased in the OFC. Given the known functions of spinophilin, increased levels likely reflect increased spine density and synaptic activity (91) following extended cocaine access. The manner in which cocaine induces such changes remains to be determined; however, a recent study has demonstrated that chronic activation of D1 and D2 receptors increase spine density and levels of spinophilin expression in striatal medium spiny neurons (92) suggesting the possibility of a similar mechanism in cortical neurons. The region specific increase in PSD-95 in the OFC is particularly interesting in light of the glutamatergic projections from this region to the nucleus accumbens in primates. DAT knockout mice exhibit reduced PSD-95 protein levels in the accumbens and targeted deletion of PSD-95 enhances LTP in frontocortical-accumbal projections (93). Assuming similar relationships in the present study (e.g., elevated DAT levels are correlated with increased PSD-95 levels), it is reasonable to suggest that OFC frontocortical-accumbal projections may exhibit reduced LTP and therefore a reduction in prefrontal regulation of accumbal function. Dysfunction of OFC – accumbal projections are thought to produce impulsivity such that current behavior is dominated by previously conditioned behaviors that may be inappropriate for normal functioning (94) – as seen in various pathological states such as addiction.

The current study provides insight into biochemical alterations occurring in prefrontal cortical regions in primate brain as a function of cocaine exposure. The changes in dopamine related proteins in prefrontal regions might contribute to alterations in the subjective experience and behavioral characteristics of chronic cocaine abusers. Examination of the effects of cocaine on cognitive functions as well as levels of receptor and transporter expression, the prefrontal circuitry primarily affected and the expression of dopamine receptor interacting proteins and proteins involved in signal transduction in the non-human primates will be fundamental to ascertaining the molecular pathology of prefrontal dysregulation in cocaine addiction.

## Authorship Credit

Leonard Howell and Scott E. Hemby designed the experiments. Scot McIntosh and Scott E. Hemby performed the experiments, collected and analyzed the data; Scott E. Hemby wrote the manuscript and Scot McIntosh and Leonard Howell provided critical review and suggestions.

## Conflict of Interest Statement

The authors declare that the research was conducted in the absence of any commercial or financial relationships that could be construed as a potential conflict of interest.

## Abbreviations

ACC, anterior cingulate cortex; DLPFC, dorsolateral prefrontal cortex; ERK, extracellular signal-regulated kinase; OFC, orbitofrontal cortex; PSD-95, post-synaptic density protein 95 kDa; TH, tyrosine hydroxylase.
